# Association of hospital volume with perioperative and long-term outcomes of minimally invasive thymectomy

**DOI:** 10.1016/j.xjon.2025.11.016

**Published:** 2025-11-24

**Authors:** Erin Yu, Li Ding, Hannah Sidhu, Graeme Rosenberg, Takashi Harano, Sean C. Wightman, Scott M. Atay, Anthony W. Kim, Brooks V. Udelsman

**Affiliations:** a Keck School of Medicine of the University of Southern California, Los Angeles, Calif; b Division of Thoracic Surgery, Department of Surgery, Keck School of Medicine of the University of Southern California, Los Angeles, Calif; c Department of Population and Public Health Sciences, Keck School of Medicine of the University of Southern California, Los Angeles, Calif

**Keywords:** minimally invasive thymectomy, high-volume centers, oncologic outcomes

## Abstract

**Background:**

Thymectomy is an uncommon procedure, and operative experience may influence outcomes. We examined the association between center-level operative volume using the National Readmissions Database (NRD) and the National Cancer Database (NCDB). These databases provide complementary insights into short- and long-term outcomes. We hypothesized that perioperative morbidity would be similar in high-volume (HV) centers and low-volume (LV) centers, but long-term survival and R0 resection—especially for minimally invasive (MI) thymectomy—would be superior in HV centers.

**Methods:**

Patients from the NRD (2017-2020) and NCDB (2010-2015 and 2018-2021) diagnosed with thymoma or thymic carcinoma who underwent thymectomy were included. (NCDB 2016-2017 was excluded for missing stage data.) The top quartile defined HV centers from LV centers. The Pearson χ^2^ test was used to calculate odds ratios (OR) and 95% confidence intervals (CIs). Logistic regression assessed factors associated with in-hospital mortality, postoperative complications, and R0 resection. Kaplan-Meier analysis evaluated long-term survival.

**Results:**

In NRD analysis (n = 3127 patients), MI thymectomy was more frequent in HV centers (57.4% vs 40.9%: *P* < .001). No differences were found in adjusted 30-day mortality (OR, 2.00; 95% CI, 0.69-5.77; *P* = .20) or postoperative complications (OR, 0.95; 95% CI, 0.76-1.19; *P* = .65). In NCDB analysis (n = 3798 patients) HV centers were associated with improved 10-year survival (hazard ratio [HR], 0.77; 95% CI, 0.61-0.97; *P* = .02) and decreased R1/R2 resection (HR, 0.77; 95% CI, 0.64-0.92; *P* = .004). MI thymectomies at HV centers were associated with a higher rate of R0 resection for stage I and stage II disease (HR, 0.47 [95% CI, 0.31-0.73; *P* = .001] and HR, 0.77 [95% CI, 0.64-0.97; *P* = .004], respectively).

**Conclusions:**

Perioperative outcomes were similar irrespective of center volume, but HV centers demonstrated improved rates of R0 resection and long-term survival even with an MI approach to thymectomy.

## Graphical Abstract

**Figure:**
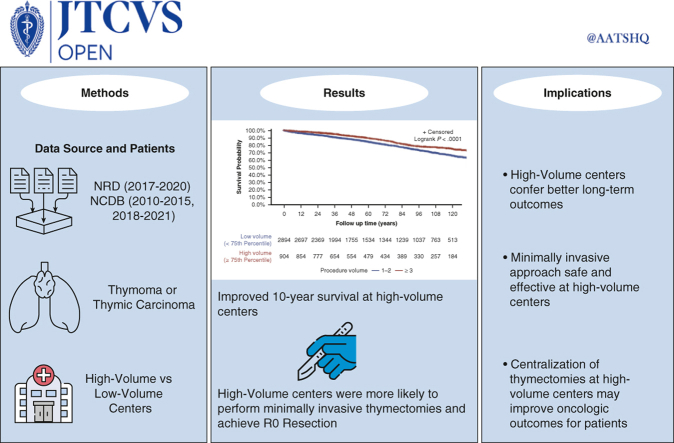

**Figure:**
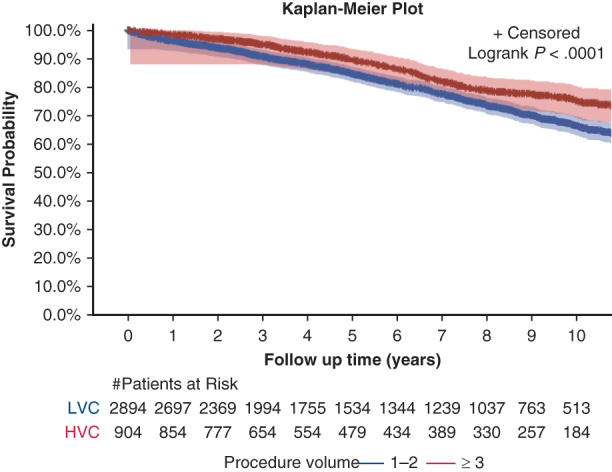
Kaplan-Meier curve of 10-year unadjusted survival between HV and LV centers. Shaded areas represent 95% confidence intervals.

**Central Message**

Minimally invasive thymectomy is associated with improved perioperative morbidity and with increased long-term survival and R0 resection rates at high-volume centers.

**Perspective**

Thymectomy is an uncommon procedure, and a center's level of experience may affect short-term and long-term outcomes in patients undergoing minimally invasive thymectomy. This study addresses the gap in knowledge as to whether center-level operative volume affects perioperative morbidity, oncologic resection, or long-term survival in patients undergoing minimally invasive thymectomy.

Rare malignant tumors of the anterior mediastinum, thymomas constitute roughly 20% of all mediastinal masses and up to 50% of all anterior mediastinal tumors, with an age-standardized incidence of 0.13 to 0.15 per 100,000 in the United States.^1^^,^^2^ Complete surgical resection (R0) remains the recommended therapy in eligible patients^3^^,^^4^; however, the role of minimally invasive (MI) techniques is a matter of debate owing to concerns of capsular disruption, tumor seeding of the pleura, incomplete resection, and local recurrence.^5^^,^^6^ Current studies have shown that MI thymectomy may be associated with less blood loss, shorter hospital stays, and similar complication rates compared to open thymectomy.^4^, ^5^, ^6^, ^7^, ^8^ Despite these perioperative advantages, however, current National Comprehensive Cancer Network (NCCN) guidelines do not routinely recommend MI procedures for tumor resection, except in selected cases of clinical stage I-II tumors when performed by experienced surgeons in specialized centers.^3^

Given the rarity of thymomas, thymectomy is a fairly uncommon operation.^1^^,^^2^ Nonetheless, with current NCCN guidelines permitting MI thymectomy when performed by experienced surgeons, understanding the influence of institutional experience is essential. This study aimed to assess the association between center-level operative volume for (1) perioperative outcomes in open and MI thymectomy using the National Readmissions Database (NRD) and (2) long-term survival using the National Cancer Database (NCDB). These complementary databases allow for a more comprehensive assessment of early- and long-term outcomes. We hypothesized that while perioperative outcomes would be similar in high-volume (HV) centers and low-volume (LV) centers, rates of R0 resection and long-term survival, especially for MI thymectomy, would be superior in HV centers.

## Methods

### Data Source

We used the National Readmissions Database (NRD) for perioperative outcomes and the National Cancer Database (NCDB) for long-term outcomes. The NRD includes approximately 16.5 million discharges annually,^9^ and the NCDB includes >34 million patient records and roughly 70% of newly diagnosed cancers.^10^ Because these datasets are not linkable, overlap between cohorts could be determined. The use of these 2 datasets provides complementary perspectives, from which we were able to evaluate short-term perioperative outcomes with the NRD and long-term oncologic outcomes using the NCDB. Both datasets are public and deidentified, and thus the requirement for informed written consent was waived by the Keck School of Medicine's Institutional Review Board (IRB #HS-23-00600; approved October 10, 2024).

### Inclusion and Exclusion Criteria

Patients from the NRD were included if they were age ≥18 years, had a diagnosis of thymoma/thymic carcinoma, and underwent thymectomy between 2017 and 2020. Analysis of the NRD defined HV centers as centers in the top quartile in operative volume (≥5 cases per year). Exclusion criteria for the NRD included nonelective cases and a resected tumor diagnosis inconsistent with a thymoma or thymic carcinoma. Nonelective cases were defined by admission through the emergency department. Patients from the NCDB were included if they were age ≥18 years, had a histology of thymoma or thymic carcinoma, and underwent thymectomy between 2010 and 2015 or between 2018 and 2021 (Tables E1 and E2). Staging for the years 2010 to 2015 was determined using clinical stages derived from the CS_EXTENSION variable, which we translated to Masaoka-Koga criteria based on prior work.^11^ The years 2016-2017 were excluded from the NCDB owing to the inadequacy of staging data collected during this period. Beginning with diagnosis year 2018, clinical staging transitioned to the American Joint Committee on Cancer TNM classification.^12^ Analysis of the NCDB defined HV centers as the top quartile in operative volume (≥3 cases per year), similar to prior studies on this topic.^11^ To ensure robustness, we conducted a sensitivity analysis using the 90th percentile cutoff of annual volume (≥10 cases/year in the NRD and ≥5 cases/year in the NCDB). Exclusion criteria for the NCDB included patients with missing data on mortality, survival, or staging and patients with stage 4 disease. Patients with missing data on stage or surgical approach were excluded from analysis because these variables were essential to the primary exposure and outcomes. For other covariates, cases with <5% missing data were retained, as prior methodological work demonstrated that such a level of missing data is unlikely to bias results.^13^

### Comparison Groups, Covariates, and Outcomes

In comparisons of HV centers and LV centers, independent variables included sociodemographic factors (age, sex, race, insurance status, income), Charlson Comorbidity Index (CCI), symptoms of myasthenia gravis, surgical approach (open vs MI), stage, neoadjuvant systemic therapy, adjuvant radiation therapy, and facility type (academic or nonacademic). Missing data for independent variables were categorized as “unknown.” The primary outcomes were perioperative morbidity, perioperative mortality, long-term survival, and rate of R0 resection. Perioperative morbidity was identified through International Classification of Diseases, 10th Revision codes as described by Kim and colleagues^14^ and included the following complications: postoperative infections, renal failure, respiratory failure, intubation, ileus, pulmonary embolism, deep vein thrombosis, postoperative shock, anemia, and wound complications (Table E3). Perioperative was defined as occurring within 30 days of the operation or within the index hospitalization. Secondary outcomes included hospital length of stay (LOS), discharge to a non-home location, and 30-day and 90-day readmissions. An independent secondary comparison of these outcomes was performed between open and MI resections. Cases that converted from MI to open procedures were classified as open.

### Statistical Analysis

Bivariate analysis of patient characteristics was performed at each cutoff for hospital volume. Statistical significance was determined using the Pearson χ^2^ test for categorical variables and the equal variance *t* test for continuous variables. For the NRD cohort, associations between hospital volume and in-hospital mortality, complications, and readmission were analyzed using logistic regression. LOS was evaluated using negative binomial regression to adjust variance inflation. Generalized estimation equations were used to adjust for the clustering effect of patients treated in the same hospital. For the NCDB cohort, LOS was analyzed using negative binomial regression to adjust for variance inflation; long-term oncologic outcomes were assessed using Kaplan-Meier survival analysis, the log-rank test, and Cox regression. The proportional hazards assumption was evaluated by Martingale residuals. R0 status was analyzed using Poisson regression with robust variance and reported as relative risk (RR).

To account for potential effects of era in the adoption of MI techniques, we also examined national trends in the use of MI thymectomy from 2010 to 2021. This approach using multivariable regression was chosen over other methodologies, such as propensity score matching for 2 primary reasons, First, both the NCDB and NRD contain some missing data, and we were concerned about violations of key assumptions and risks of introducing additional bias.^15^ Additionally, the primary exposure of interest was a hospital-level characteristic (center volume) rather than a patient-level variable, and as a result, only a very limited set of variables could be incorporated into a propensity model, making this approach less appropriate in our study context. All statistical tests were 2-sided, with significance defined as *P* < .05. Analyses were conducted using SAS version 9.4 (SAS Institute).

## Results

### Patient Demographics

**Figure 1:**
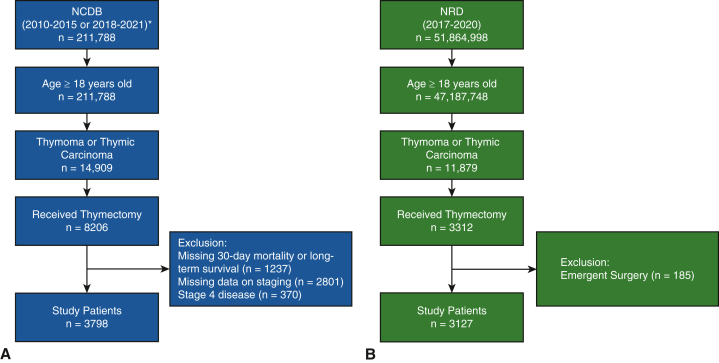
Inclusion and exclusion criteria for the National Cancer Database (NCDB) (A) and Nationwide Readmissions Database (NRD) (B) cohorts. ∗The years 2016-2017 were excluded due to inadequacy of collected staging data during this period.

The NRD was used to evaluate perioperative outcomes in a total of 1877 patients who received care at HV centers and 1250 patients treated at LV centers (Figure 1). Patients at HV centers had higher CCI scores (*P* < .001) and higher income status (*P* < .001) compared to patients at LV centers (Table 1). HV centers also were more likely to be teaching hospitals (96.3% vs 80.8%; *P* < .01). MI thymectomy was more common at HV centers than at LV centers (57.4% vs 42.6%; *P* < .001).

**Table 1:** Demographic data for the NCDB and NRD cohorts

Characteristic	NCDB			NRD		
	LV centers (N = 2894)	HV centers (N = 904)	*P* value	LV centers (N = 1250)	HV centers (N = 1877)	*P* value
Age, y, mean (SD)	60.0 (13.67)	59.7 (13.34)	.560	57.3 (15.21)	56.2 (15.27)	.043
Sex, n (%)			.227			.052
Male	1424 (49.2)	424 (46.9)		570 (45.6)	790 (42.1)	
Female	1470 (50.8)	480 (53.1)		680 (54.4)	1087 (57.9)	
CCI, n (%)			.147			<.001
0	2136 (73.8)	696 (77.0)		609 (48.7)	804 (42.8)	
1	568 (19.6)	153 (16.9)		238 (19.0)	325 (17.3)	
≥2	190 (6.6)	55 (6.1)		403 (32.3)	748 (39.8)	
Stage, n (%)			.377			
I	1877 (64.9)	573 (63.4)		-	-	
II	428 (14.8)	151 (16.7)		-	-	
III	589 (20.4)	180 (19.9)		-	-	
Insurance, n (%)			<.001			<.001
Medicare	1145 (40.1)	303 (34.2)		474 (38.0)	625 (33.3)	
Medicaid	228 (8.0)	38 (4.3)		159 (12.7)	184 (9.8)	
Private	1334 (46.7)	527 (59.4)		549 (44.0)	1003 (53.5)	
Other	148 (5.2)	19 (2.1)		66 (5.3)	64 (3.4)	
MG symptoms, n (%)	-	-		256 (20.5)	376 (20.0)	.760
Surgery route, n (%)			<.001			<.001
Minimally invasive	979 (33.8)	407 (45.0)		511 (40.9)	1077 (57.4)	
Open	1915 (66.2)	497 (55.0)		739 (59.1)	800 (42.6)	
Income, n (%)			<.001			<.001
Quartile 1	222 (8.8)	32 (4.0)		286 (23.0)	316 (17.0)	
Quartile 2	390 (15.4)	101 (12.5)		335 (27.0)	388 (20.9)	
Quartile 3	677 (26.8)	154 (19.1)		327 (26.3)	484 (26.1)	
Quartile 4	1237 (49.0)	518 (64.3)		293 (23.6)	666 (35.9)	
Teaching hospital, n (%)	986 (37.3)	750 (90.3)	<.001	1010 (80.8)	1807 (96.3)	<.001
Race, n (%)			<.001			
White	2095 (73.0)	619 (69.6)		-	-	
Black	513 (17.9)	125 (14.0)		-	-	
Asian	205 (7.1)	119 (13.4)		-	-	
Other	55 (1.9)	27 (3.0)		-	-	
Tumor size, cm, mean (SD)	6.90 (6.05)	6.43 (4.21)	.093	-	-	
Neoadjuvant therapy, n (%)	188 (6.5)	113 (12.5)	<.001	-	-	
Adjuvant radiotherapy, n (%)	903 (31.2)	235 (26.0)	.003	-	-	

*NCDB*, National Cancer Database; *NRD*, National Readmissions Database; *LV*, low volume; *HV*, high volume; *SD*, standard deviation; *CCI*, Charlson Comorbidity Index; *MG*, myasthenia gravis.

The NCDB was used to evaluate long-term outcomes in a total of 904 patients who received care at HV centers and 2894 treated at LV centers (Table 1). Patients at HV centers had higher percentages of private insurance (59.4% vs 46.7%; *P* < .001) and higher quartile income (64.3% vs 49.0%; *P* < .001). Similar to the NRD, the NCDB cohort had a higher rate of MI thymectomy at HV centers compared to LV centers (45.0% vs 33.8%; *P* < .001). The mean tumor size was for HV and LV centers were 6.43 (SD, 4.21) cm at HV centers and 6.90 (6.05) cm at LV centers (*P* = .093). HV centers were more likely than LV centers to administer neoadjuvant systemic therapy (12.5% vs 6.5%; *P* < .001) and less likely to administer adjuvant radiation therapy (26.0% vs 31.2%; *P* = .003). In both databases, HV centers showed increased use of MI techniques, and the adoption of these techniques increased over time. HV centers also were more likely to be teaching hospitals, indicating that trends in volume and institutional characteristic were consistent across databases (Table 1).

### Perioperative Outcomes

Perioperative outcomes were assessed within the NRD between 2017 and 2020 in a total of 3127 patients. In-hospital mortality rates were similar between HV and LV centers, with in-hospital mortality rates of 0.32% and 0.96%, respectively (*P* = .36), and this was maintained in adjusted analysis (OR, 2.00; 95% CI, 0.69-5.77; *P* = .20) (Table 2). Mortality rates also were not significantly different between HV and LV centers (0.67% vs 0.97% [*P* = .4] at 30 days and 0.89% vs 1.75% [*P* = .07] at 90 days). Postoperative complications also did not differ by case volume, as HV centers reported an OR of 0.95 compared to LV centers (95% CI. 0.76-1.19; *P* = .65). Other perioperative outcomes, such as hospital LOS, discharge to non-home, and 30-day and 90-day readmissions, did not differ significantly by center volume; however, postoperative complications were more likely after open thymectomy compared to MI thymectomy (OR, 3.00; 95% CI, 2.40-3.74; *P* < .001). When using a 90th percentile cutoff for the NRD, associations between HV centers and perioperative outcomes remained nonsignificant for complications, LOS, discharge, and 30-day and 90-day admissions (Table E4).

**Table 2:** Adjusted perioperative outcomes using the NRD

Outcome	LV centers (N = 1250)	HV centers (N = 1877)	*P* value
In-hospital mortality, OR (95% CI)	Ref.	2.00 (0.69-5.77)	.20
Postoperative complications, OR (95% CI)	Ref.	0.95 (0.76-1.19)	.65
Discharge to non-home, OR (95% CI)	Ref.	0.93 (0.73-1.19)	.56
30-d readmission, OR (95% CI)	Ref.	0.88 (0.63-1.24)	.47
90-d readmission, OR (95% CI)	Ref.	0.96 (0.72-1.28)	.79
Length of stay, IRR (95% CI)	Ref.	0.99 (0.90-1.10)	.89

	MI thymectomy (N = 1588)	Open thymectomy (N = 1539)	
Postoperative complications, OR (95% CI)	Ref.	2.30 (2.40-3.74)	<.001
Discharge to non-home, OR (95% CI)	Ref.	3.37 (2.98-4.67)	<.001
30-d readmission, OR (95% CI)	Ref.	1.32 (0.94-1.85)	.11
90-d readmission, OR (95% CI)	Ref.	1.27 (0.96-1.67)	.09
Length of stay, IRR (95% CI)	Ref.	2.21 (2.02-2.41)	<.001

Separate multivariable logistic regression models were constructed for each outcome, with adjustment for the same set of covariates. Outcomes for postoperative complications, discharge, readmission, and length of stay were adjusted for the confounders of procedure volume, age, sex, Charlson Comorbidity Index, insurance, myasthenia gravis symptoms, surgery route, income, and teaching status of hospitals.

*LV*, Low volume; *HV*, high volume; *OR*, odds ratio; *CI*, confidence interval; *IRR*, incidence rate ratio; *MI*, minimally invasive.

### Long-Term Outcomes

Long-term outcomes were assessed in the NCDB for 2010-2015 and for 2018-2021, which included a total of 3798 patients. Adoption of MI techniques increased across the NCDB study window, with 46 cases (3.3%) performed in 2010, compared to 421 cases (30.4%) performed in 2021. In adjusted analysis, patients from HV centers exhibited lower odds of mortality at 10 years compared to those from LV centers (HR, 0.77; 95% CI, 0.61-0.966; *P* = .02) (Table 3). Patients who underwent open thymectomy exhibited a 47.5% higher risk of mortality at 10 years compared to recipients of MI thymectomy (HR, 1.48; 95% CI, 1.22-1.79; *P* < .001). When stratified by both stage and surgical method, open thymectomy performed at HV centers compared to LV centers had better 10-year survival rates for stage 1 disease (81.1% vs 70.2%; *P* = .005), as well as for stage 2 disease (80.5% vs 63.0%; *P* = .005) (Table 4). In addition to 10-year survival, 5-year survival was evaluated using adjusted analysis and proved to be significantly different between the HV and LV centers (*P* < .001). Patients treated at LV centers had a lower 5-year survival (compared to those treated at HV centers (84.8% [SE, 0.152] vs 89.5% [SE, 0.012]; *P* < .001).

**Table 3:** Adjusted 10-year mortality using the NCDB

Mortality	LV centers (N = 2894)	HV centers (N = 904)	*P* value
10-y mortality, HR (95% CI)	Reference	0.77 (0.61-0.97)	.02

	MI thymectomy (N = 1386)	Open thymectomy (N = 2412)	
10-y mortality, HR (95% CI)	Reference	1.48 (1.22-1.79)	<.001

Separate multivariable logistic regression models were constructed for each outcome, with adjustment for the same set of covariates. Outcomes were adjusted for the confounders of procedure volume, age, sex, race, Charlson-Deyo score, insurance, surgical approach, tumor stage, income, hospital teaching status, neoadjuvant and adjuvant therapy, and year of diagnosis.

*LV*, Low volume; *HV*, high volume; *HR*, hazard ratio; *CI*, confidence interval; *MI*, minimally invasive.

**Table 4:** Adjusted 10-year survival rate by stage and surgical technique

Stage, technique	10-y survival rate, % (95% CI)		*P* value
	LV centers (N = 2894)	HV centers (N = 904)	
Stage 1, open	70.2 (66.5-73.9)	81.1 (74.2-88.0)	.005
Stage 1, MI	76.2 (70.1-82.3)	81.5 (74.1-88.9)	.226
Stage 2, open	63.0 (53.8-72.2)	80.5 (71.7-89.3)	.005
Stage 2, MI	76.3 (70.2-82.4)	74.6 (59.3-89.9)	.563
Stage 3, open	54.1 (48.0-59.2)	60.4 (51.6-69.2)	.276
Stage 3, MI	59.0 (44.7-73.3)	63.0 (37.9-88.1)	.260

Outcomes were adjusted for the confounders of procedure volume, age, sex, race, Charlson Comorbidity Index, insurance, income, hospital teaching status, neoadjuvant and adjuvant therapy, and year of diagnosis.

*CI*, Confidence interval; *LV*, low volume; *HV*, high volume; *MI*, minimally invasive.

Rates of R0 resection were evaluated amongst patients in HV versus LV centers. In adjusted analysis, patients at HV centers had a decreased risk of undergoing R1/R2 resection (RR, 0.77; 95% CI, 0.674-0.92; *P* = .004) (Table 5). When stratified by stage and surgical method, treatment at HV centers conferred a significantly lower risk of R1/R2 resection for patients who underwent MI thymectomy in stage 1 disease (RR, 0.47; 95% CI, 0.31-0.73; *P* = .001), and in stage 2 disease (RR, 0.77; 95% CI; 0.64-0.92; *P* = .004). For patients with stage III disease, there was no significant increase in the rate of R0 resection at HV centers for MI thymectomy (RR, 0.84, 95% CI 0.49-1.44; *P* = .530) or open thymectomy (RR, 0.87; 95% CI, 0.62-1.21; *P* = .410). At a higher cutoff of the 90th percentile of cases in the NCDB, HV centers were associated with significantly improved 10-year survival (HR, 0.66; 95% CI, 0.47-0.95; *P* = .023) and a lower risk of incomplete resection (RR, 0.76; 95% CI, 0.58-0.99; *P* = .047) (Table E4). In multivariable analysis, disease stage was independently associated with receipt of MI thymectomy, with stage 1 patients significantly more likely to undergo MI resection compared to stage 2 patients (RR, 0.93; 95% CI, 0.90-0.96; *P* < .001) and stage 3 patients (RR, 0.88; 95% CI, 0.85-0.91; *P* < .001).

**Table 5:** Adjusted risk of incomplete resection by stage and surgical technique

Stage, technique	Incomplete resection, RR (95% CI)		*P* value
	LV centers (N = 2894)	HV centers (N = 904)	
Overall	Reference	0.77 (0.64-0.92)	.004
Stage 1, open	Reference	0.83 (0.55-1.27)	.395
Stage 1, MI	Reference	0.47 (0.31-0.73)	.001
Stage 2, open	Reference	0.61 (0.33-1.14)	.120
Stage 2, MI	Reference	0.77 (0.64-0.92)	.004
Stage 3, open	Reference	0.87 (0.62-1.21)	.410
Stage 3, MI	Reference	0.84 (0.49-1.44)	.530

Outcomes were adjusted for the confounders of procedure volume, age, sex, race, Charlson Comorbidity Index, insurance, income, hospital teaching status, neoadjuvant and adjuvant therapy, and year of diagnosis.

*RR*, Relative risk; *CI*, confidence interval; *MI*, minimally invasive.

Finally, there was a transition of staging systems during the study period. A sensitivity analysis for the years 2010-2015 using the Masaoka-Koga system and for 2018-2021 using the eighth edition TNM showed consistently higher rates of R0 resection for 2018-2021 (RR, 0.78; 95% CI, 0.62-0.97; *P* = .025 for 2010-2015; RR, 0.71; 95% CI, 0.51-0.97; *P* = .033 for 2018-2021) (Table E5). For 10-year survival, HV centers were associated with significantly improved outcomes in Masaoka-Koga stage (HR, 0.75; 95% CI, 0.59-0.97; *P* = .03), but not in the eighth edition TNM stage (HR, 0.71; 95% CI, 0.41-1.24; *P* = .23).

## Discussion

**Figure 2:**
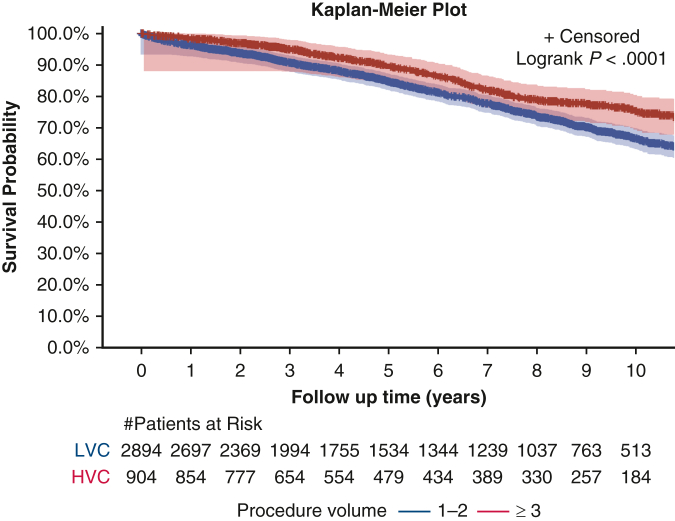
Kaplan Meier curve of 10-year unadjusted survival between high-volume centers (HVC) and low-volume centers (LV) with 95% confidence intervals.

In this dual-database retrospective analysis of patients undergoing thymectomy, treatment at HV centers was associated with improved long-term outcomes and increased use of MI thymectomy compared to LV centers. Although perioperative outcomes did not differ significantly by center volume, long-term survival and rate of R0 resection were notably superior at HV centers (Figure 2).

Patients treated at HV centers had higher incomes and more private insurance and were more often treated at teaching hospitals. These factors may reflect the benefits of broader institutional resources and multidisciplinary capabilities that contribute to favorable outcomes for complex procedures, although we could not evaluate the effect of individual surgeon expertise, given the lack of surgeon-level identifiers in our dataset.^16^, ^17^, ^18^ Nevertheless, these results mirror those reported by Birkmeyer and colleagues: patients requiring complex oncologic surgery tend to do better at high-volume centers.^19^ While MI techniques were more common at HV centers, the benefit of treatment at these centers is likely multifactorial and due to potential differences in adjuvant treatment, follow-up, tumor biology, and institutional experience treating these rare tumors rather than to surgical technique alone. This is supported by improved survival for open thymectomies for stage I and II disease seen in HV centers compared to LV centers.

Despite similar short-term outcomes, HV centers conferred significant long-term survival benefit, with a 23.2% lower hazard of 10-year mortality in multivariable analysis. Importantly, MI thymectomy was independently associated with fewer postoperative complications and improved long-term survival. Prior studies have reported equivalent R0 resection rates and 5-year survival rates for MI thymectomy and open thymectomy.^19^^,^^20^ When stratified by disease stage and surgical approach, our study showed that HV achieved higher R0 resection rates, particularly in patients undergoing MI thymectomy for early-stage disease. Our findings align with a recent study demonstrating that treatment at HV centers was associated with higher odds of R0 resection and improved 5-year and 10-year survival.^11^ Taken together, these results support the hypothesis that institutional experience contribute to long-term survival and oncologic efficacy, and that MI thymectomy is not associated with worse oncologic outcomes at HV centers. The current study supports NCCN guidelines advocating for reservation of MI approach HV centers.

Interestingly, HV centers were more likely to use neoadjuvant systemic therapy and less likely to administer adjuvant radiation, suggesting more complete resections and optimized perioperative care. The observed survival advantage at HV centers supports regionalization of thymic surgery to HV institutions with experience in MI techniques and access to comprehensive cancer care. While a potential downside to regionalization is limiting access to treatment, we believe that these modest cutoffs are unlikely to result in significant additional burden.^21^, ^22^, ^23^, ^24^ Further research is needed to confirm this in thymectomy.

An unexpected finding was the increase in long-term survival among early-stage patients undergoing open thymectomy at HV centers. Despite classification as stage I or II, technical challenges due to proximity to vital mediastinal structures may result in the decision to undergo an open approach, allowing for controlled dissection in anatomically complex cases. The observed survival benefit for open thymectomy in early-stage disease may reflect the expertise, resources, and multidisciplinary support available at HV centers. These findings further highlight the benefits of receiving care for early-stage thymomas at HV centers irrespective of the surgical approach.

Strengths of this study include use of 2 large complementary databases providing the power to study both short- and long-term outcomes for a rare procedure. Limitations include residual confounding factors despite adjustment and a lack of data on recurrence, referral patterns, surgeon case volume, and clinical factors such as frailty and pulmonary function. We also confirmed in multivariable analysis that earlier-stage disease was independently associated with receipt of MI thymectomy, highlighting stage as an important potential confounder when interpreting survival outcomes. While we performed robust multivariable analysis to control for differences in patient populations, it remains possible that despite our robust statistical power, these confounders effected the results. Anatomic details, such as tumor proximity to critical structures and the extent of resection, were not captured, limiting our ability to assess the appropriateness of surgical approaches in complex cases.

Likewise, we grouped thymoma with thymic carcinoma in our analysis, given the extreme rarity of thymic carcinoma. Considering the complexity of management and need to for multidisciplinary approach, we strongly advocate for treatment of thymic carcinoma at highly specialized centers. Data on the type of MI approach were limited, and we were not accurately or consistently able to distinguish between the types of MI approaches (eg, substernal, transcervical, transthoracic). How the type of MI approach may have influenced the primary outcomes and the role of surgeon preference are unclear. The NCDB does not capture disease-specific survival, so we were unable to distinguish between mortality related to thymic malignancy and mortality from other causes. Although we used 2 large national databases, differences in variable definitions and follow-up periods between the NRD and NCDB may limit direct comparisons, and there may be overlaps in some of the patient population. In addition, analyses of stage III patients were likely underpowered given the small sample size, which may explain the absence of significant differences in R0 resection in this subgroup. During the study period, the NCDB transitioned from Masaoka-Koga staging to eighth edition TNM staging, which might have confounded the results; however, in sensitivity analysis separating the respective staging time periods, the improved R0 resection rates remained. The later cohort did not reach statistical significance for 10-year survival, possibly owing to limited follow-up duration, smaller sample size, and lower power to detect differences. Finally, despite the use of national databases in this analysis, thymic diseases remain rare, and some trends may have lacked sufficient statistical power to reach significance.

## Conclusions

**Figure 3:**
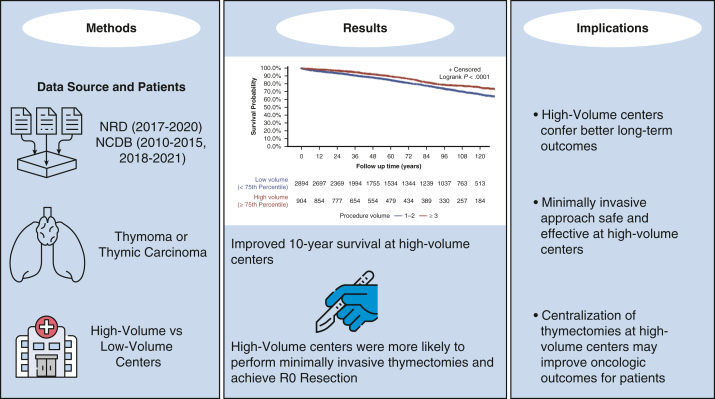
Graphical abstract.

HV centers are associated with significantly improved long-term survival and R0 resection (Figure 3). Benefits of greater institutional experience were evident for both open and MI approaches, as well as for early-stage disease. These findings provide retrospective observational support for the selective referral of thymic surgery to HV centers. Although the findings do not indicate causality, they do warrant future prospective studies to elucidate institutional and surgical experience behind these volume–outcome relationships and to establish best practices for these rare thoracic malignancies.

## Conflict of Interest Statement

The authors reported no conflicts of interest.

The *Journal* policy requires editors and reviewers to disclose conflicts of interest and to decline handling or reviewing manuscripts for which they may have a conflict of interest. The editors and reviewers of this article have no conflicts of interest.

## Figures and Tables

**Table E1:** ICD-O3 codes used in the NCDB

ICD-O-3 code	Histologic term(s)	Primary site
858	Thymic epithelial neoplasms	C37.9
8580/0	Thymoma, benign	C37.9
8580/1	Thymoma, NOS	C37.9
8580/3	Thymoma, malignant, NOS	C37.9
8581/1	Thymoma, type A, NOS; Thymoma, medullary, NOS; Thymoma, spindle cell, NOS	C37.9
8581/3	Thymoma, type A, malignant; Thymoma, medullary, malignant; Thymoma, spindle cell, malignant	C37.9
8582/1	Thymoma, type AB, NOS; Thymoma, mixed type, NOS	C37.9
8582/3	Thymoma, type AB, malignant; Thymoma, mixed type, malignant	C37.9
8583/1	Thymoma, type B1, NOS; Thymoma, lymphocyte-rich, NOS; Thymoma, lymphocytic, NOS; Thymoma, organoid, NOS; Thymoma, predominantly cortical, NOS	C37.9
8583/3	Thymoma, type B1, malignant; Thymoma, lymphocyte-rich, malignant; Thymoma, lymphocytic, malignant; Thymoma, organoid, malignant; Thymoma, predominantly cortical, malignant	C37.9
8584/1	Thymoma, type B2, NOS; Thymoma, cortical, NOS	C37.9
8584/3	Thymoma, type B2, malignant; Thymoma, cortical, malignant	C37.9
8585/1	Thymoma, type B3, NOS; Thymoma, atypical, NOS; Thymoma, epithelial, NOS	C37.9
8585/3	Thymoma, type B3, malignant; Thymoma, atypical, malignant; Thymoma, epithelial, malignant; Well-differentiated thymic carcinoma	C37.9
8586/3	Thymic carcinoma, NOS; Thymoma, type C	C37.9

*ICD-O-3*, International Classification of Diseases for Oncology, Third Edition; *NOS*, not otherwise specified.

**Table E2:** ICD-10 diagnosis codes used in the NRD

Diagnosis	ICD-10 code
Neoplasm of thymus	D15.0
Persistent hyperplasia of thymus	E32.0
Disease of thymus, unspecified	E32.9
Malignant neoplasm of thymus	C37
Malignant carcinoid tumor of the thymus	C7A.091

*ICD-10*, International Classification of Diseases, 10th Revision.

**Table E3:** ICD-10 diagnosis codes for postoperative complications using the NRD

Category	Complication	ICD-10 code
Infectious		
	Postoperative infection	T8140XA
	Postoperative septic shock, initial encounter	T8112XA
	Postoperative skin infection	T8149XA
	Postoperative Subdiaphragmatic abscess	
	Postoperative infected seroma (Postprocedural Seroma of Skin and SubQ Tissue Following other Procedure)	L7634
Genitourinary		
	Renal Failure (Postprocedural [acute] [chronic] kidney failure)	N99
	Urinary Tract Infection, site not specified	N39
	Other Postprocedural complications and disorders of GU System	N9989
	(Postoperative Urinary Retention and Urinary Tract Infection)	
Pulmonary		
	Ventilator-Associated Pneumonia	J95851
	Other Postprocedural complications and disorders of Respiratory System, NEC (not elsewhere classified)	J9589
	Other Complication of Respiratory (Ventilator)	J9588
	Other Postoperative complications of respiratory system, not elsewhere classified	J9589
	Acute Postprocedural Respiratory Failure	J95821
	Acute Respiratory Failure, Unspecified Whether with Hypoxia or Hypercapnia	J9600
	Acute Pulmonary Insufficiency Following Thoracic Surgery	J951
	Chronic Pulmonary Insufficiency Following Surgery	J953
	Acute and Chronic Postprocedural Respiratory Failure	J95822
	Acute and Chronic Respiratory Failure, Unspecified Whether with Hypoxia or Hypercapnia	J9620
	Postprocedural Pneumothorax	J95811
	Postprocedural Air Leak >5 d	J95.812
	Postprocedural Hemorrhage of a respiratory system organ or structure following a respiratory system procedure	J95.830
	Acute Pulmonary Edema	J810
	Postprocedural Hematoma of a Respiratory System Organ or Structure following a Respiratory System Procedure	J95.860
	Postprocedural Seroma of a Respiratory System Organ or Structure following a Respiratory System Procedure following a Respiratory System Procedure	J95.862
	Emphysema (subcutaneous) resulting from a procedure (Initial Encounter)	T81.82XA
Gastrointestinal		
	Postprocedural Intestinal Obstruction (Ileus)	K913
Cardiovascular		
	Septic Pulmonary Embolism without Acute Cor Pulmonale	I2690
	Other Pulmonary Embolism without Acute Cor Pulmonale	I2699
	DVT (Complication of Vein following Procedure, not elsewhere classified, initial encounter)	T81.72XA
	Acute Embolism and Thrombosis of unspecified deep veins of lower extremity	I82.40
	Posthemorrhagic anemia, acute blood loss anemia, acute hemorrhage anemia, acute loss of blood anemia, acute postoperative anemia, acute anemia secondary to blood loss	D62
Systemic		
	Postprocedural Shock Unspecified, Initial Encounter	T8110XA
	Postprocedural Cardiogenic Shock, Initial Encounter	T8111XA
	Postprocedural Septic Shock, Initial Encounter	T8112XA
	Other Postprocedural Shock, Initial Encounter	T8119XA
	Other Complications of Procedure not Elsewhere Classified, Initial Encounter	T8189XA
	Unspecified Complication of Procedure, Initial Encounter	T819XXA
Other Wound-Related Complications		
	Other Specified Complications of Surgical and Medical Care, not Elsewhere Classified, Initial Encounter	T888XXA
	Disruption of Wound, Unspecified, Initial Encounter	T8130XA
	Disruption of Internal Operation (Surgical) Wound, not Elsewhere Classified, Initial Encounter	T8132XA
	Disruption of External Operation (Surgical) Wound, not Elsewhere Classified, Initial Encounter	T8131XA
	Disruption of Traumatic Injury Wound Repair, Initial Encounter	T8133XA
	Persistent Postprocedural Fistula, Initial Encounter	T8183XA
	UTI (Other Postprocedural complications and disorders of GU System)	N99.89
	Surgical Site Infection (Infection following a Procedure, Superficial Incisional Surgical Site) (“”, Deep Incisonal Surgical Site) (Initial Encounter)	T81.41XA, T81.42XA
	Sepsis (following a procedure)	T81.44XA
	Surgical Site Complications	T81.1-T81.49
	Arterial, venous, lung, and air embolisms	I26.0, I26.9, and I80 (all)
Complications of Procedures, not Elsewhere Classified		
	Postprocedural Shock	T81.1
	Disruption of Wound, NEC	T81.3
	Infection Following a Procedure	T81.4
	Vascular Complications Following Procedure	T81.7
	Other Complications of Procedures	T81.8
	Unspecified Complication of Procedure	T81.9

*ICD-10*, International Classification of Diseases, 10th Revision; *GU*, genitourinary; *NEC*, necrotizing enterocolitis.

**Table E4:** Sensitivity analysis of hospital volume cutoffs

NRD	LV centers (<10 cases/year)	HV centers (≥10 cases/year)	*P* value
Postoperative complications, OR (95% CI)	Reference	0.94 (0.72-1.22)	.625
LOS, OR (95% CI)	Reference	1.07 (0.93-1.23)	.358
Discharge, OR (95% CI)	Reference	1.06 (0.78-1.46)	.698
30-d readmission, OR (95% CI)	Reference	1.08 (0.76-1.52)	.679
90-d readmission, OR (95% CI)	Reference	1.05 (0.78-1.41)	.749

NCDB	LV centers (<5 cases/year)	HV centers (≥5 cases/year)	
10-y mortality HR (95% CI)	Reference	0.66 (0.47-0.95)	.023
Incomplete resection RR (95% CI)	Reference	0.76 (0.58-0.99)	.047

Outcomes were adjusted for the confounders of procedure volume, age, sex, race, Charlson Comorbidity Index, insurance, surgical approach, tumor stage, income, hospital teaching status, neoadjuvant and adjuvant therapy, and year of diagnosis.

*NRD*, National Readmissions Database; *LV*, low volume; *HV*, high volume; *OR*, odds ratio; *CI*, confidence interval; *NCDB*, National Cancer Database; *HR*, hazard ratio; *RR*, relative risk.

**Table E5:** Sensitivity analysis of staging transition

Outcomes of HV centers vs LV centers	2010-2015 (Masaoka-Koga) (N = 2163), HR (95% CI)	2018-2021 (8th ed TNM) (N = 1635), HR (95% CI)
10-y mortality	0.754 (0.585-0.973); *P* = .03	0.711 (0.408-1.239); *P* = .23
Incomplete R0 resection	0.777 (0.623-0.969); *P* = .025	0.706 (0.513-0.972); *P* = .033

Outcomes were adjusted for the confounders of procedure volume, age, sex, race, Charlson Comorbidity Index, insurance, income, hospital teaching status, neoadjuvant and adjuvant therapy, and year of diagnosis.

*HV*, High volume; *LV*, low volume; *HR*, hazard ratio; *CI*, confidence interval.
